# The partner’s experience of traumatic brain injury and its recovery

**DOI:** 10.2217/cnc-2016-0012

**Published:** 2016-07-28

**Authors:** Gerard A Riley

**Affiliations:** 1 School of Psychology, University of Birmingham, Edgbaston, Birmingham B15 2TT, UK

**Keywords:** caregiver, family, partner, spouse, traumatic brain injury

Traumatic brain injury (TBI) presents significant challenges to the partner of the person with the TBI and to the relationship they share. Struggling to deal with those challenges can have a significant impact on the psychological well-being of the partner, and on the quality and stability of the relationship. Compared with the general population, partners generally report higher levels of anxiety, stress and depression [1,2]; and lower levels of positive well-being, quality of life and life satisfaction [1,2]. Although there may be improvements [2], these difficulties can persist for many years after the TBI [3]. In terms of the impact on the relationship, methodological inadequacies in the research mean that there is, as yet, no clear answer to the question of whether TBI increases the rate of marital breakdown compared with the general population [4]. However, on measures of overall marital quality and satisfaction, partners tend to give their current relationship a poorer rating in comparison to the general population and to the preinjury relationship [4,5].

The primary aim of this overview is to summarize research about the partner’s negative experiences of TBI that may explain this damaging impact that the injury can have on their psychological well-being and the relationship. Understanding the experiences that contribute to the impact will highlight issues that need to be addressed in supporting couples after a TBI. The overview describes experiences both in the initial stages and in the longer term. It addresses the partner’s experience of more severe TBI (i.e., one that requires hospitalization and has long-lasting effects) rather than mild TBI. It should be noted that, although all the research papers cited in the review included at least some participants who were the partners of someone with a more severe TBI, a substantial number used mixed samples that contained participants who had other forms of brain injury (e.g., stroke), other familial relationships with the person with the injury (e.g., parents) or experience of mild TBI. Some partners have more positive experiences of TBI and this research, which is more limited in extent, will also be summarized. Understanding positive experiences may shed light on why some partners cope better than others.

## Experiences prior to discharge from the hospital

The initial response of partners to TBI is one of shock, concern about how severe the injury is and, in some cases, fear about whether the person will survive [6]. Uncertainty about the prognosis and likely impact of the injury can be stressful and many partners report a strong need for information and explanation [6,7]. Once the person begins to improve, there can be a sense of relief and optimism about further progress [2].

Relationships with hospital staff are sometimes difficult. Partners may feel that they are not being given all the information they need; that they are not adequately involved in decisions and that their knowledge of the injured person is not taken into proper account [7]. They may feel emotionally unsupported by clinicians [7]. They may also be concerned about the quality of care provided. Inconsistent advice from different professionals may be interpreted as incompetence and leads to mistrust in the ability of the hospital staff to provide the best care [8]. There may be conflict with clinicians about what are perceived by clinicians as the partner’s unrealistic expectations about recovery, and clinicians’ predictions about progress that turn out to be incorrect can lead to anger and mistrust [7]. When the injured person is transferred from acute to rehabilitation services, partners may be disappointed with the amount and quality of rehabilitation [6,7]. Some partners report feeling generally disappointed, angry and frustrated with the hospital system [8].

In some cases, the injured person may fail to regain full consciousness and spends many months in the hospital. Some partners feel regret and guilt about decisions that have prolonged a life of little quality, rather than allowing the person to die, or guilt about wishing the person was dead [9]. They may feel stressed and exhausted as they continue their hospital visits at the same time as managing their employment and family commitments; and other aspects of their life may suffer from the lack of time and energy they have to maintain them [9–11]. Grief over the ‘loss’ of the injured person and their life together is common, and this may be difficult to resolve because the person is lost in one sense, but still present in another [10,11]. Difficult relationships with clinicians may persist, focusing on the same issues as those outlined in the previous paragraph [9,10].

## Experiences related to discharge from the hospital

Discharge from the hospital can be associated with mixed emotions. On the one hand, there may be a sense of optimism and excitement at the prospect of helping the person recover [6,7,12]. There may also be relief that the stresses associated with the period in hospital are at an end and that life can get back to normal [6,7,12]. On the other hand, there may be worries about how the injured person will cope and what the future holds for them; about the partner’s own ability to provide care and support for the person and about the arrangements for community support and rehabilitation [6]. These concerns may be heightened by a perception that clinicians have not provided adequate advice or guidance on how to manage the injured person, or on how to access and arrange community support and rehabilitation [8]. They may also be heightened by an increasing awareness of the disabilities arising from the TBI now that the person is out of the protective hospital environment in which not all of their disabilities were apparent [12].

Another source of distress is the realization of the limited nature of community support and rehabilitation [6–8,13,14]. Some partners report that they feel abandoned and unsupported by services in the immediate aftermath of discharge, left to cope by themselves with little support or guidance at a time when their responsibilities and need for assistance have increased [13]. The task of arranging support and rehabilitation sometimes falls on partners and this can prove a frustrating experience: they may lack knowledge of what is available or what the injured person needs; much effort and time may be required to arrange services and communication between different services may be inadequate, resulting in poor coordination of service delivery [6–8,13,14].

## Experiences of living with TBI in the longer term

### Extra responsibilities, roles & tasks

A major source of physical and emotional strain for partners is the fact that, in addition to all of the responsibilities, roles and tasks that they had prior to the TBI, they now have to take on many additional ones involved in the care-giving role. The person with the injury may need assistance with a range of activities of daily living such as self-care and mobility [3]. The partner may have to take on, or assume a larger part in, some everyday tasks and roles previously done by the injured person – for example, child care and managing household finances [5,15,16]. Additional demands on their time include those related to the ongoing rehabilitation of the injured person, such as taking them to hospital appointments, helping them carry out rehabilitative exercises at home and encouraging their engagement in activities [13]. The partner often has a significant role to play in providing emotional support to the injured person [12,13,17]. In some cases it may not be safe to leave the injured person on their own and constant supervision may be required [18]. Unsurprisingly, the partner can feel burdened, exhausted and overwhelmed by all these extra demands [3,13,15,17].

### Neglect of their own needs & interests

For many partners, dealing with these extra demands means that they have little time to pursue their own personal interests and activities, or to address their own needs [11,13,15,17]. Some may reduce or give up their own employment and career plans in order to support the person with the injury [11]. Time spent on leisure and social activities may also be very limited [7,11,13,17]. The plans and goals that they had for their own lives may be viewed as being ‘put on hold’ or ‘lost’, and there may be feelings of frustration and sadness about this [17]. Some partners report that the focus of others (e.g., family, friends, clinicians and the injured person) is on the person with the TBI and not on the partner [13,15]. Together with the sacrifice of their own interests and needs, this can lead to a sense that their individuality has been compromised and a feeling that they are just a care-giver with no life or identity separate from this role [14,17].

### Financial difficulties

Financial difficulties can be a source of stress [10,15]. The partner and/or the injured person may have to move to less well-paid employment, reduce their employment hours or give up paid employment altogether [6,14,15]. There may also be added costs associated with ongoing medical and rehabilitation needs.

### Dealing with behavioral & personality changes

A consistent research finding is that partners are particularly upset by some of the personality and behavioral changes that may result from TBI, in the form of either the appearance of undesirable behaviors such as aggression and social disinhibition, or the loss of beneficial attributes such as motivation [14,16,18,19]. Different changes can elicit different emotional responses in the partner. Lack of initiative and motivation, for example, can give rise to frustration, anger and resentment [18,20]. Generally, the loss of beneficial attributes may be associated with a sense of sadness and loss when a contrast is drawn with how the person used to be [18]. Aggression, on the other hand, can lead to a state of perpetual and fearful vigilance, as the partner watches for signs and triggers of the aggression and tries to forestall its occurrence [18]. Partners can also feel angry about the aggression and the impact it has [18]. Difficulties in understanding why the person is behaving in these ways can lead to frustration and bewilderment [14,18], and lack of success in managing the behaviors can lead to a sense of helplessness and hopelessness [14,16,18].

One particular category of beneficial attributes that may be diminished or lost are those involved in positive social interaction. TBI may cause problems in reading social signals and communications from others, in empathizing and understanding the needs and perspective of the other person, in expressing positive emotions and in communicating generally. In the context of an intimate relationship, these problems may show themselves in the form of an apparent coldness, indifference and lack of affection [18,20]. The injured person may appear self-centered and unresponsive to the needs of the partner, showing little awareness or appreciation of how the partner is feeling [18,20]. Conversation, shared enjoyment and humor may be limited [20]. Partners can find this lack of emotional warmth and responsiveness particularly upsetting, and it may lead to feelings of dissatisfaction, loneliness and a sense of loss for the affection and warmth they used to receive [5,15,18–20].

### Relationship with the injured person

TBI can have a negative impact on the relationship between the partner and the person with the injury. The changes in personality and behavior described in the previous section can create in the partner a perception that the identity of the injured person has undergone a radical transformation: The injured person may feel like a stranger who is no longer recognizable as the same person that they lived with before the injury, and whose new personality they have to learn to understand [15,17–19]. Together with changes in roles and the need to provide care to the injured person, this experience of living with a stranger can also contribute to a feeling that the relationship itself is radically changed; and that it has become more like a parent/child or caregiver/care–recipient relationship, rather than a marriage/partnership [5,15,17–19]. Together with the changes in personality and behavior, these experiences of radical change in identity and the relationship can, in turn, have a negative impact on the love that the partner feels toward the injured person. Love may be replaced by other feelings such as those associated with a protective and caring role, or, in some cases, outright dislike [5,15,18–20]. Some partners describe feelings of loss for the preinjury person they no longer have, for the preinjury relationship and for the life that they shared together [14,16–20].

The partner’s wish for sexual intimacy may diminish. Intimacy can be difficult with someone whom one no longer loves, who feels like a stranger, appears cold and indifferent or is prone to aggression [15,18,19]. The provision of personal care, and the experience of being in a caregiver/care–recipient relationship, can also interfere with sexual feelings [5]. The injured person may also have sexual dysfunction or other physical difficulties as a result of the injury that interfere with the sexual aspects of the relationship [15].

In the face of all of these changes, some partners feel ambiguous about remaining in the relationship [14,18,19]. Those who do remain may do so out of a sense of obligation. The resulting conflict between wanting to leave and feeling that they ought to stay can make them feel trapped, resentful and angry. Thoughts about leaving can, in turn, give rise to feelings of guilt [14]. Partners may also feel guilty because of the anger, resentment and other negative feelings that they experience toward the person with the TBI; and because of how they sometimes behave toward the injured person [14,17,18].

### Other relationships within the family

Some consequences of TBI, such as aggression and difficulties in engaging in positive social interaction, can have an impact on other relationships within the family and on the general functioning of the family which, in turn, can increase the stress on the partner [15]. The partner may be required to manage relationships between the injured person and other family members (particularly children), fostering good relationships, preventing and repairing fractures to relationships and in some cases, protecting others from harm [14–16]. Some partners worry about the longer term impact of the TBI on their children, a worry fueled by their concern that they may be neglecting the needs of their children because of the extra demands placed on them by the injured person [14,15,17]. The partner may also have to provide information, explanations and emotional support to others within the family to help them to cope with what has happened [13]. Such provision may be more challenging when they are required by members of the extended family who do not live within the family home: They may not fully appreciate the impact of the TBI on the person with the injury and may, in consequence, have misguided and hurtful attitudes toward the partner [13,15]. Contact with the wider family may sometimes diminish because of such attitudes, because the wider family avoids the injured person due to difficulties in coping with their behavior, or because the partner does not have time to cultivate and maintain those relationships because of the extra demands placed on them [14,15,17].

### Relationships with friends & the wider community

For various reasons, relationships with friends and the wider community can become restricted and some partners report feeling socially isolated and lonely [17]. As noted earlier, because of the extra demands, the partner may reduce or give up employment and may limit time spent on leisure and social activities [7,11,13,17]. Behavioral changes in the injured person may be a source of embarrassment when with friends or in public, and some partners may restrict social contact because of this [14,16–18]. Friends and members of the public may also react to the injured person in negative ways that upset the partner and make them reluctant to expose the person with TBI to such contact [7,12,13]. Friends may be reluctant to maintain contact with the family because they do not feel comfortable around the injured person and struggle to cope with the changes they perceive [17].

### Facing the challenges alone

Some partners describe feeling alone in facing the challenges posed by the brain injury and by life in general [15,17,19]. As described earlier, they may feel abandoned by clinical services once the injured person is discharged from the hospital [13], contact with the extended family, friends and the wider community may diminish and support from family and friends may be limited [14,15,17]. The partner may no longer feel able to confide in, or seek support from, the person with the TBI, partly because of their disabilities and partly because of their apparent self-centeredness and unresponsiveness to the needs of the partner [15,17,19,20].

## Individual differences & positive experiences

The focus of this overview has been on the negative experiences of TBI that may undermine the psychological well-being of the partner and their relationship with the person with the TBI. Partners, of course, vary greatly in their experience of TBI [20]. Whether they have these negative experiences, and the extent to which they have them, differs between partners. Not all partners report clinical levels of anxiety or depression, or decreased levels of life satisfaction and well-being [1,2]. Not all experience a decline in the quality of their relationship or lose their love for the injured person [4,15,18]. Some cope effectively with behavioral and personality changes [18]. Many report good relationships with clinicians and are satisfied with the rehabilitation services they receive [14], and many feel well supported by family and friends [11,17].

Indeed, for some partners, it is not just that they are less affected by these negative experiences; the TBI also brings some positive experiences. These include the rewards of helping and supporting someone you love, and the satisfaction of seeing progress and improvement [14,17]. Some partners have also reported personal growth and development as a result of meeting the challenges posed by the injury, including a new appreciation of what life offers, and the development of inner strength and resilience [11,14]. Some have also reported that dealing with the injury has strengthened their relationship with the injured person and brought them closer together [5,14,15,18].
